# Influence of Annealing and Biaxial Expansion on the Properties of Poly(l-Lactic Acid) Medical Tubing

**DOI:** 10.3390/polym11071172

**Published:** 2019-07-11

**Authors:** Brian Dillon, Patrick Doran, Evert Fuenmayor, Andrew V. Healy, Noel M. Gately, Ian Major, John G. Lyons

**Affiliations:** 1 Materials Research Institute, Athlone Institute of Technology, Dublin Road, Bunnavally, Athlone, N37 HD68 Co. Westmeath, Ireland; 2 Faculty of Engineering and Informatics, Athlone Institute of Technology, Dublin Road, Bunnavally, Athlone, N37 HD68 Co. Westmeath, Ireland

**Keywords:** bioabsorbable polymers, poly-l-lactic acid, annealing, biaxial expansion, orientation, crystallinity, strain rate

## Abstract

Poly-l-lactic acid (PLLA) is one of the most common bioabsorbable materials in the medical device field. However, its use in load-bearing applications is limited due to its inferior mechanical properties when compared to many of the competing metal-based permanent and bioabsorbable materials. The objective of this study was to directly compare the influence of both annealing and biaxial expansion processes to improve the material properties of PLLA. Results showed that both annealing and biaxial expansion led to an overall increase in crystallinity and that the crystallites formed during both processes were in the α’ and α forms. 2D-WAXS patterns showed that the preferred orientation of crystallites formed during annealing was parallel to the circumferential direction. While biaxial expansion resulted in orientation in both axial and circumferential directions, with relatively equal sized crystals in both directions, Da (112 Å) and Dc (97 Å). The expansion process had the most profound effect on mechanical performance, with a 65% increase in Young’s modulus, a 45% increase in maximum tensile stress and an 18-fold increase in strain at maximum load. These results indicate that biaxially expanding PLLA at a temperature above T_cc_ is possible, due to the high strain rates associated with stretch blow moulding.

## 1. Introduction

Poly-l-lactic acid (PLLA) is an aliphatic polyester derived from renewable resources. Due to its biodegradability and biocompatibility, it is one of the most studied and used bioabsorbable materials in the medical device field. Its primary degradation mechanism is through hydrolysis, which results in the subsequent loss of molecular weight, strength and eventually mass, before being metabolised into carbon dioxide and water via the Krebs cycle [1,2]. PLLA is a semi-crystalline polymer, with a glass transition temperature (*T*_g_) of 60–65 °C and melting temperature (*T*_m_) of 175–180 °C. Despite having reasonably good mechanical strength compared to other bioabsorbable polymers, with a tensile strength of 60–70 MPa and elastic modulus of 2–4 GPa, it is limited in its use due to its brittle nature with less than 5% strain and poor fracture toughness [3]. It also has inferior mechanical properties compared to many of the competing metal-based permanent and bioabsorbable materials used in similar load-bearing medical applications, such as scaffolding, orthopaedics and fixation devices.

As with most polymers, the mechanical properties of PLLA is heavily influenced by molecular weight and crystallinity [1,4,5]. The degree of crystallinity of a bioabsorbable polymer is directly related to its hardness, modulus, tensile strength, stiffness, hydrophilicity, degradation rate and melt temperature [6]. While PLLA is a semi-crystalline polymer, traditional melt processing techniques, such as extrusion and injection moulding, result in a mostly amorphous material due to PLLA’s slow crystallisation kinetics [7]. PLLA crystals are formed through different thermal and mechanical treatments. It can crystallise in three structural forms, known as α (alpha), β (beta), γ (gamma), which are distinguishable by different helix conformations and cell symmetries. The most stable and common form is the α form, and its growth is attributed to melting or cold crystallisation (*T*_cc_), typically at a crystallisation temperature (*T*_c_) greater than 120 °C. The α form also takes a more disordered form, known as the α’, which is formed at a lower *T*_c_, typically less than 90 °C. There is, however, an α’-α transition between 90–120 °C, where the less stable α’ transitions to the more stable α form [8,9]. The β form is developed from high draw rates and high-temperature mechanical stretching of the α form [10], with the γ form developed on hexamethylbenzene substrates [11].

There has been extensive research into the use of poly(lactic acid) (PLA) blends and composites to improve bulk properties [12,13,14]. The inclusion of fillers, such as silica [15,16,17], carbon nanotubes [18], natural fibres [19] and nanoclays [20,21], have all had reported success in improving the materials’ thermal stability, mechanical performance and degradation rate. However, limited clinical data and questions, remaining over the bioresorbability and processability (dispersion) of many of these nanocomposites, has hindered their emergence into mainstream commercial applications. Therefore, to improve the mechanical properties of PLLA, secondary processing techniques, such as annealing [22,23,24,25,26] or biaxial expansion [27,28,29,30], have been the preferred method employed by commercial medical device manufacturers.

The annealing or sometimes referred to as isothermal treatment of PLLA above its glass transition temperature results in an increase in mechanical properties owed to a thickening of the lamella and increase in overall crystallinity [28,31,32,33]. Both annealing temperature and time play a key role in defining the materials crystalline structure [25,34,35,36]. While the biaxial expansion of PLLA causes strain-induced orientation and crystallisation, which increases the mechanical strength and toughness [29,37]. PLLA’s slow crystallisation kinetics are enhanced through mechanical deformation, resulting in strain-induced molecular orientation [38,39,40,41]. During expansion, the degree of strain (expansion ratio), temperature, deformation mode (simultaneous or sequential) and strain rate are all key to controlling the degree of crystallisation and molecular orientation imparted in the material [29,42,43,44]. It has been reported that to achieve strain-induced crystallisation in PLLA, the degree of strain must be >100% when expanded at a temperature just above *T*_g_ [45]. However, as the expansion temperature increases, so does the minimum required degree of strain [39]. Strain-induced crystallisation also requires the strain rate to be equivalent to or higher than chain relaxation time. Chain relaxation is caused by chain retraction and is strongly affected by expansion temperature. Typically, an expansion temperature between *T*_g_ and *T*_cc_ (>65 and <100 °C) is selected, as below *T*_g_, there isn’t enough chain mobility to induce crystallisation, and at temperatures approaching or above *T*_cc_, chain relaxation is faster than the draw rate [45].

The effect that both annealing and biaxial expansion exerts on the properties of PLLA has been extensively researched. However, there have been limited studies, which directly compare the influence of both processes on PLLA tubing. Also, most of the previous work has been performed at expansion temperatures between *T*_g_ and *T*_cc_, and at low strain rates <4 s^−1^ [39,41,42,45,46]. This is despite the strain rates associated with a traditional stretch blow moulding process used for tube expansion being significantly higher, up to 15 s^−1^ [47]. These higher strain rates result in the requirement of higher expansion temperatures to enable strain-induced crystallisation [28]. It has also been reported that temperatures in the range of 100–130 °C have been found to produce Expanded tubes with more uniform wall thickness and stiffness, as well as improved optical clarity [48]. The aim of this study was to directly compare the influence of both secondary processes for the enhancement of properties in PLLA tubing for load bearing medical applications. This involved using two separate annealing cycles and a stretch blow moulding process for biaxial expansion, with its associated high strain rate and expansion temperature.

## 2. Materials and Methods

### 2.1. Materials and Processing

The material used was a poly-l-lactic acid (PLLA), which was obtained from Corbian (Purac, Amsterdam, The Netherlands). It was medical grade homopolymer of l-lactide with the chemical formula (C6H8O4). It was a medical grade homopolymer of l-lactide with a molecular weight of Mn 325,000 and Mw 650,000. For the purpose of this study, Extruded tubing was manufactured on a 1” extruder (American Kuhne, York, PA, USA) to the outside diameter (OD) of 1.9 mm and wall thickness (WT) of 0.6 mm [49]. Post-extrusion, the tubing was then either Annealed or biaxially Expanded. The annealing process utilised a Sanyo Gallenkamp (Loughborough, UK) annealing oven, with the tubing subjected to one of two different annealing cycles, 70 °C for 60 min (Annealed70) or 100 °C for 60 min (Annealed100). The biaxial expansion process involved the simultaneous radial and axial expansion of the raw Extruded tubing using an in-house built stretch blow moulding machine as previously described [48]. During expansion, the polymer was heated to a temperature of 115 °C and expanded at a strain rate of 12 s^−1^. The blow-up ratio (BUR) and axial draw ratio (ADR) of the Expanded tubes are detailed below:

(1) $$\frac{OD\;of\;the\;expanded\;tube}{ID\;of\;extruded\;tube}=\;\frac{3.25\;mm}{0.71\;mm}=4.57\;BUR$$

(2) $$\frac{Length\;of\;the\;expanded\;tube}{Length\;of\;extruded\;tube}=\;\frac{217\;mm}{100\;mm}=2.17\;ADR$$

### 2.2. Thermal and Morphological Properties

#### 2.2.1. Differential Scanning Calorimetry

Differential Scanning Calorimetry (DSC) was used to assess the thermal and morphological properties of the PLLA Extruded, Annealed and Expanded tubing. Testing was performed using a DSC 2920 instrument (TA Instruments, New Castle, DE, USA). The DSC thermograms were obtained by heating the samples (8–12 mg) from 30 to 250 °C at a rate of 10 °C/minute under a nitrogen atmosphere. Representative thermograms were acquired from three samples (*n* = 3) tested in each group and are presented with their standard deviation (σ) in Table 1. The influence of thermal history during processing was assessed by comparing the first heating cycle of the various samples. The following thermal properties were analysed; specific heat change (Δ*C*_p_), glass transition temperature (*T*_g_), melt recrystallisation temperature (*T*_mc_), melting temperature (*T*_m_), cold crystallisation temperature (*T*_cc_), enthalpy of melting (Δ*H*_m_) and enthalpy of cold crystallisation (Δ*H*_c_) from the first heating cycle of the various samples. The degree of crystallinity (*X*_c_) was calculated using the enthalpy (heat) of melting Δ*H*_m_ and the enthalpy of cold crystallisation Δ*H*_c_ (where present), through the following equation:

(3) $$X_{c}\;\left(\%\right)\;=\;\frac{\Delta H_{m}\;-\;\Delta H_{c}}{\Delta {H_{m}}^{0}\;}\;\times \;100$$

where a Δ*H_m_*^0^ = 93 J/g is used as the enthalpy of fusion for a 100% crystalline PLLA [50].

#### 2.2.2. Wide Angle X-Ray Scattering

Wide Angle X-Ray Scattering (WAXS) analysis was performed on a 3-circle, Bruker D8 Quest single crystal diffractometer (Bruker AXS Inc, Madison, WI, USA) equipped with a microfocus copper source (Kα = 1.54184 Å) and a Photon 2 detector. The samples were manually aligned along the fixed κ angle (54.8°) and ω set at 0°. All measurements were obtained at room temperature in air and analysed in the 2θ range of 5–40 degrees. The data was integrated with the Debye ring tool in the Bruker Apex 3 software and analysed in the Panalytical X’Pert Highscore Plus. The mean crystal size (D_hkl_) of the ordered (crystalline) domains was calculated from the Debye-Scherrer equation, expressed as:

(4) $$D_{\mathrm{hkl}}\;=\frac{K\lambda }{\beta_{1/2}\cos\theta }$$

D_hkl_ was calculated for both the circumferential (Dc) and axial (Da) directions and was found at the (110)/(200) plane with a scattering angle 2θ of 16.3–16.4°. K is a dimensionless shape factor (0.9), λ is the X-ray wavelength (1.54184 Å), β is the width at half the maximum of the peak height (FWHM) for the selected Bragg diffraction angle (θ) [51].

### 2.3. Mechanical Properties

#### 2.3.1. Tensile Testing

The tensile properties of the various samples were evaluated using a Lloyd LRX tensometer, equipped with a 2.5 kN load cell (Bognor Regis, UK). Samples were tested at ambient temperature with an extension rate of 50 mm/minute and a gauge length of 65 mm. The Young’s (elastic) modulus, maximum tensile stress and strain at maximum load were averaged over five samples (*n* = 5) and are presented with their standard deviation (σ) in Table 2. Testing was completed in accordance with the International Organization for Standardization (ISO) 527 standard. The Extruded, Annealed70 and Annealed100 tubing had a part geometry consisting of a 1.9 mm OD and 0.60 mm WT. The Expanded tubing had a part geometry of 3.25 mm OD and 0.115 mm WT.

#### 2.3.2. Dynamic Mechanical Analysis

The thermo-mechanical analysis was performed using TA Instruments DMA (Dynamic Mechanical Analysis) Q800 (New Castle, DE, USA). The samples were heated at a ramp rate of 5 °C/minute, between a temperature range of 0 to 140 °C, with a frequency of 1 Hz and an amplitude of 15 μm in tension mode using a TA Instruments standard tension film clamp. Representative DMA plots of both the storage modulus and tan delta were acquired from three samples (*n* = 3) tested in each group, with the average storage modulus and associated standard deviation (σ) presented Table 3. As the samples were tested in their tubular form, direct compliance to an American Society for Testing and Materials (ASTM)/ISO standard was not possible. Therefore, the results presented are deemed comparative only, and general adherence to ASTM D5026 was applied except for part geometry and sample preparation. The sample part geometry (OD and WT) was identical to that listed in Section 2.3.1, with a tube (gauge) length of 16 mm.

## 3. Results and Discussion

### 3.1. Thermal and Morphological Results

#### 3.1.1. Differential Scanning Calorimetry (DSC)

**Figure 1:**
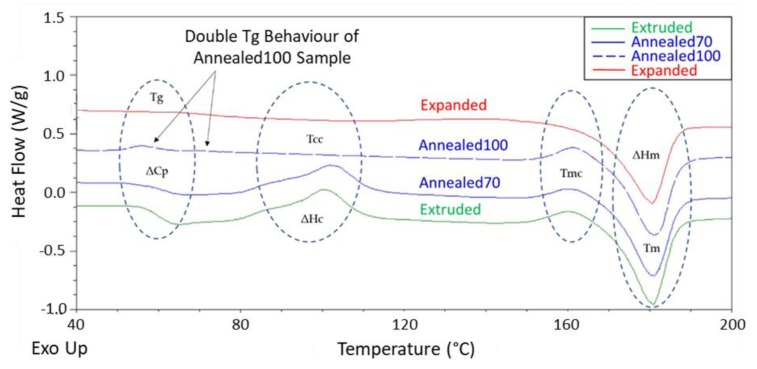
Differential Scanning Calorimetry (DSC) thermogram overlay of Extruded, Annealed70, Annealed100 and Expanded tubing. Abbreviations: T_g_: glass transition temperature; ΔC_p_: specific heat change; T_cc_: cold crystallisation; ΔH_c_: enthalpy of cold crystallisation; T_mc_: melting recrystallisation; T_m_: melting temperature; ΔH_m_: enthalpy of melting; X_c_: the degree of crystallinity.

Thermal analysis using differential scanning calorimetry (DSC) was carried out on Annealed and Expanded tubing, to determine the effect different processing conditions had on the thermal properties and X_c_ of the material. For comparison, the raw Extruded tubing was also analysed. The 1st heating cycle from the DSC analysis was used to identify any differences in thermal history between the various samples. Figure 1 provides a thermogram with an overlay of the Extruded, Annealed70, Anealed100 and Expanded tubes, while Table 1 presents a summary of their key thermal properties and X_c_.

The thermograms of both the Extruded and Annealed70 tubing are very comparable, where it is noticeable that the annealing process had little effect on the material’s thermal properties or *X*_c_. Both samples displayed a *T*_g_ in the region of 61 °C, which is shown in Figure 1 as a jump in the heat capacity (Δ*C*_p_), as evident by the baseline change in heat flow. This was followed by the presence of a broad double exothermic peak just after *T*_g_, which was again present in both samples. This peak related to *T*_cc_ and could be seen at a temperature of ~101 °C. Cold crystallisation was often found in polymers that are quench cooled during melt processing and having slow crystallisation kinetics. There was also a smaller exothermic peak just before *T*_m_, which was related to melting recrystallisation (*T*_mc_). A more detailed description of both *T*_cc_ and *T*_mc_ has been reported by the authors in a previous study into the effect of extrusion on the properties of PLLA [49]. The large endothermic peak was the melting temperature (*T*_m_), which resulted from the melting of the crystalline structure at ~181 °C. The area under the *T*_cc_ and *T*_m_ peaks is known as the enthalpy of cold crystallisation (Δ*H*_c_) and the enthalpy of heating (Δ*H*_m_), respectively, and from which the degree of *X*_c_ was calculated. Both the Extruded and Annealed70 tubing had relatively low *X*_c_, in the range of 13–14%.

**Figure 2:**
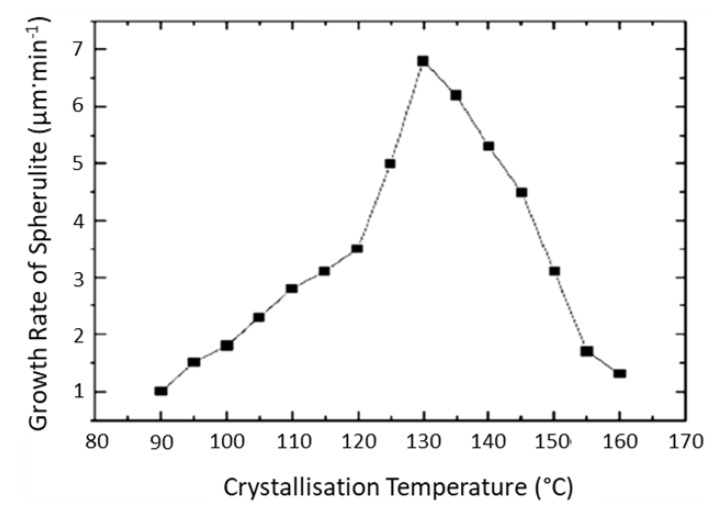
Spherulite growth rates of Poly-*l*-lactic acid (PLLA), isothermal crystallisation after cooling from the melt as a function of temperature [24] under open access license.

The minimal effect of the Annealing70 cycle on the thermal properties of the Extruded tubing was owed to the spherulite growth rate versus crystallisation temperature for PLLA, as shown in Figure 2. From the plot, it can be seen that the rate of crystallisation increases as a function of temperature and that maximum crystal growth happens at 130 °C, with a slower rate of crystal growth in the α’-α transition range of 90–120 °C [24]. The effect of annealing PLLA moulded and extruded samples have been widely studied [22,25], with one study publishing analogous results to those reported above, when injection moulded amorphous samples were annealed at 70 °C for 1 h [23].

**Figure 3:**
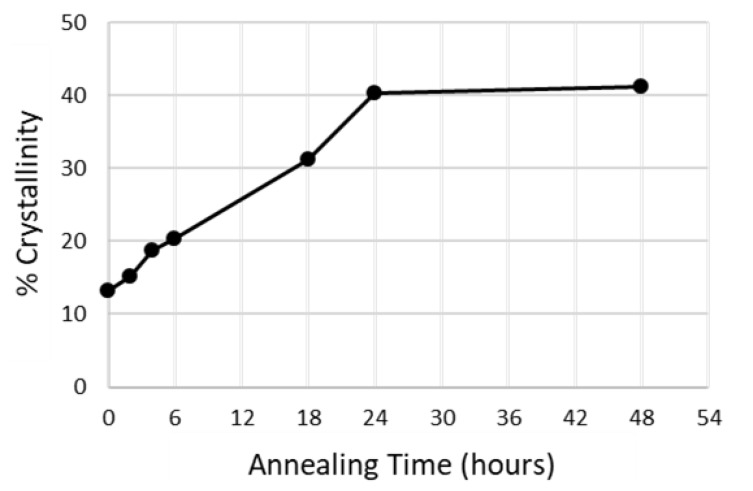
Change in crystallinity Poly-l-lactic acid (PLLA) Extruded tubing annealed at 70 °C as a function of annealing time.

It has also been reported that the degree of crystallisation in PLLA is a function of both temperature and time. A constant crystallinity is achieved at the early stages (10–15 min) of annealing, and longer annealing times do not result in an increase in crystallinity and should be avoided due to the increased risk of thermal degradation [33]. However, that study only observed the effect of annealing temperatures in the range of 100 to 160 °C. Figure 3 plots the change in crystallinity (measured by DSC) of Extruded tubing annealed at 70 °C during a 48-h period. The graph shows a linear increase in crystallinity over the first 24 h before a plateau is reached at ~40% crystallinity after 24 h. A similar increase in crystallinity over longer annealing times (hours) has been reported previously [23].

**Figure 4:**
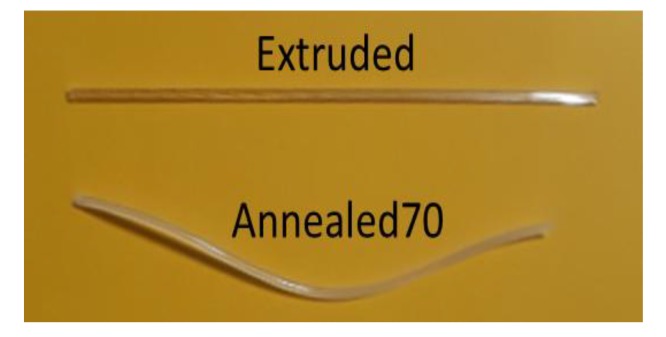
Image of Extruded and Annealed70 tubing, highlighting the level of curvature in the tube post-annealing.

One observation noted during annealing was the amount of curvature in the tubing upon removal from the annealing oven. Figure 4 shows an image of a curved tube removed from the oven after the Annealed70 cycle and, for comparison, an Extruded tube showing no curvature. The risk with annealing PLLA at temperatures above its *T*_g_ is found when frozen in stresses in part, relieved during annealing, can cause shrinkage or warpage [25]. The straightness and dimensional uniformity of Extruded tubing is a critical factor in most medical tubing applications. One option to resolve this issue would be to insert mandrels into the tubing prior to annealing. However, this could significantly increase the manufacturing cycle time.

Several observations from the thermal analysis of the Expanded and Anealed100 tubing can be made. Firstly, there was a 12–15 °C increase in the *T*_g_ and ~75% reduction in the specific heat change (ΔC_p_) of the Expanded tubing when compared to the Extruded and Annealed70 tubing. This increase in *T*_g_ and reduction in Δ*C*_p_ is associated with a reduction in the amorphous regions of the material. The biaxial expansion process has led to an increase in crystalline regions and subsequent decrease in amorphous regions within the polymer, therefore, reducing the amount of specific heat change associated with the second order glass transition (*T*_g_). One anomaly in the results is the *T*_g_ of the Annealed100 tubing, which at 63.2 °C appears lower than expected, despite the significant increase in crystallinity. This resulted in a Δ*C*_p_ higher than that of the Expanded tubing. On closer examination, there is evidence of a double *T*_g_ peak, which is skewing the temperature to the lower end and influencing the Δ*C*_p_ values. This double *T*_g_ behaviour has been attributed to partial relaxation of the rigid amorphous phase [52].

There was no evidence of cold crystallisation present in the Annealed100 tubing, due to a lack of a *T*_cc_ exothermic peak. This is due to annealing at the higher temperature, which provided enough molecular mobility for greater crystallisation, resulting in the elimination of cold crystallisation upon re-heating from the glassy state. In a study investigating the effect of thermally-induced crystallisation of PLLA, a similar elimination of cold crystallisation was reported [32]. However, there was still a small exothermic melt recrystallisation peak (*T*_mc_) before *T*_m_, meaning there was still some level of crystal perfecting or α’-α transition at this annealing temperature.

The biaxial expansion of the PLLA tubes also resulted in the elimination of the exothermic T_cc_ peak, as well as the *T*_mc_ peak. This corresponded in a ~265% increase in the degree of crystallisation when compared to the Extruded and Annealed70 tubing, with an 18% increase when compared to the Annealed100 tubing. Previous studies have reported similar findings [27,28,30], which highlight the strain-induced crystallisation capacity of PLLA. It has also been reported that the rate of crystallisation is faster in stretched PLLA than quiescent cold crystallisation at the same temperature [46]. The disappearance of *T*_mc_ in the Expanded tubing is owed to the strain-induced α’ crystal being relatively more perfect than the annealed samples, coupled with the fact that the α’ to α transition is constrained by the strain-induced molecular orientation during DSC testing [53]. It is also worth noting the influence prior melting conditions can have on the thermal properties of PLLA and how different heating and cooling rates applied during DSC analysis can significantly affect crystallisation behaviour [54]. The X_c_ in the Expanded tubing can be tuned for specific applications by optimisation of the expansion temperature, rate and ratio.

#### 3.1.2. Wide Angle X-Ray Scattering (WAXS)

**Figure 5:**
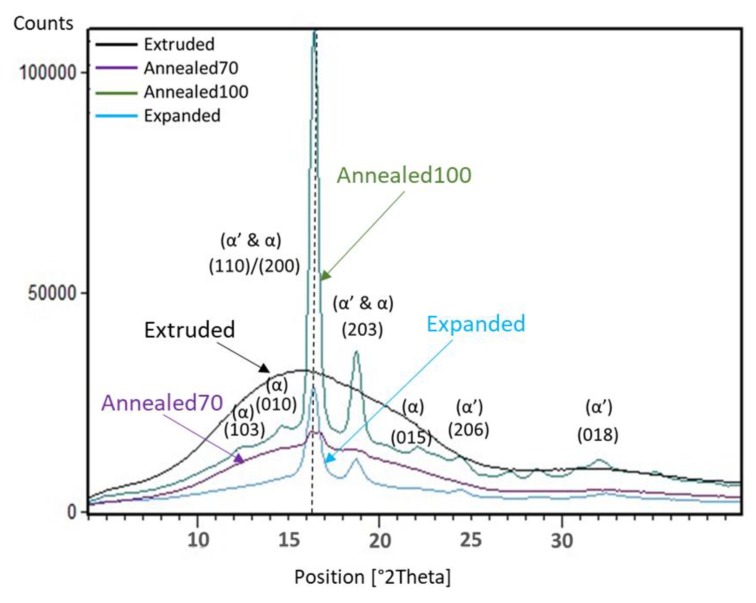
Wide Angle X-Ray Scattering (WAXS) diffractogram overlay of Extruded, Annealed70, Annealed100 and Expanded tubing.

WAXS analysis was used to look at the difference in crystallinity and degree of orientation between the various samples. It has been reported that the X-ray diffraction peaks for PLLA occur in multiple planes, with the most common reflections for the more stable α crystal form, including the (103), (010), (110)/(200), (203) and (015) planes. The less stable α’ crystal also has reflections for the (110)/(200) and (203) planes, but at slightly lower 2θ positions [29,55], with additional reflections of (206) and (018) found at higher 2θ positions [29,56,57]. Figure 5 presents an overlay of representative WAXS diffractograms from each sample, with both the Extruded and Annealed70 tubes displaying a broad diffraction peak, characteristic of a mostly amorphous material. This correlated with the low X_c_ results found during DSC testing, as presented in Table 1. However, the Annealed70 tube displayed a smaller area under the broad amorphous peak. This is most likely attributable to an increase in the transitional or mesophase between the amorphous and the crystalline phases, caused by the low-temperature annealing process. The Annealed100 tube had high-intensity reflections at (110)/(200) and (203), with smaller reflections at the (103), (010), (015), (206) and (018) planes, indicative of both the α’ and α crystal forms. This was to be expected, given an α’-α phase transition occurs between a temperature range of 90–120 °C, where the less stable α’ transitions to the more stable α form [8,9].

The Expanded tube also had high-intensity reflections at (110)/(200) and (203), with very minor reflections at the (206) and (018) planes. The lack of reflections at the (103), (010), (015) planes, would indicate the crystal formed during biaxial expansion is also in the α’-α transition phase. It’s also been reported that reflections for the (110)/(200) planes occur at lower 2θ positions in strained PLLA samples compared to those ISO thermally treated. This has been attributed to the strain-induced crystals having a larger interplanar spacing [29,39]. However, the overlay in Figure 5 shows that both the Annealed100 and Expanded tubing have almost identical 2θ positions 16.3–16.4°. This is most likely caused by the expansion temperature being greater than the T_cc_, which permits greater chain mobility, resulting in a decrease in interplanar spacing as the crystals are packed more closely [53].

**Figure 6:**
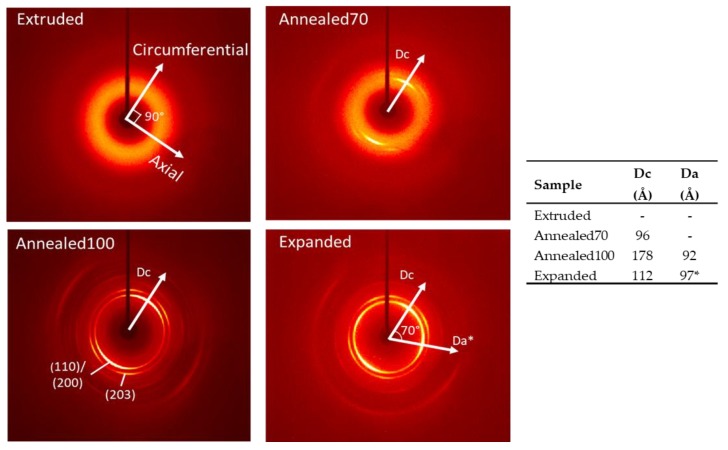
2D-WAXS (Wide Angle X-Ray Scattering) patterns of the Extruded, Annealed70, Annealed100 and Expanded tubing, plus the calculated crystal size in circumferential (Dc) and axial (Da) directions. Note: The Da* direction in the Expanded tube is offset 70° to the Dc direction.

The 2D-WAXS patterns in Figure 6 show the Extruded tube as a diffuse amorphous halo. For reference, the Extruded pattern also includes the orientation of both the axial and circumferential directions, which are perpendicular to each other. In the Annealed70 sample, two very weak equatorial diffraction peaks, which represented the (110)/(200) planes, could be found parallel to the circumferential direction. These peaks indicated the preferred orientation of the mesocrystals and α’ crystals present in the sample, which was transverse to the draw direction from the original extrusion process. The intensity of the two equatorial diffraction peaks in the Annealed100 pattern was noticeably stronger. As discussed above, this was due to the increase in X_c_ at higher annealing temperature. As expected, the preferred orientation of the Annealed100 tube was also parallel to the circumferential direction. There is a noticeable difference in the Expanded tubing, with the emergence of two additional equatorial diffraction peaks. The formation of these additional peaks corresponded with the radial straining during the simultaneous biaxial expansion process. Interestingly, the peaks were not fully parallel to the axial direction, with a 70° offset from the circumferential direction. It has been reported that the expansion of PLLA at high draw temperatures, results in a tilting of the crystal chain axis away from the draw direction. This is attributed to chain retraction being faster than the draw rate, which indicates that the orientation of the crystal nuclei is influenced by the network relaxation processes [45].

The diffractogram of the Annealed100 tube displayed a higher degree of crystallinity than the Expanded tube. This is evident by the higher intensity peak of the (110)/(200) plane at the 16.4° 2θ position. The (110)/(200) peak width was also narrower, with an FWMH of 0.66° in the Annealed100 tube compared to 0.72° in the Expanded tube. The FWMH is related to crystallite dimensions, with narrower half-widths corresponding to larger crystallites. This relationship differs from the DSC results presented in Section 3.2.1., which indicated that the Expanded tubing had a higher X_c_. However, differences in crystallinity obtained from DSC and XRD were to be expected. The diffractograms from XRD focused on the crystallites present in the material and were taken from the sample surface and, therefore, were more strongly influenced by processing conditions, such as strain-induced orientation and surface cooling. Alternatively, DSC measured the enthalpy of heating from a bulk sample. It has also been suggested that heating during DSC could potentially affect the materials’ crystallinity [23].

Using the Debye-Scherrer equation, the crystal size in circumferential (Dc) and (where applicable) axial (Da) directions were measured. The Extruded tube wasn’t calculated, given its mostly amorphous morphology, however, the Annealed70 tube had a Dc of 96 Å. The Annealed100 crystal size was measured in both the circumferential (Dc) and axial (Da) directions, with the Dc (178 Å), being twice as large as the Da (92 Å). It had been previously reported that simultaneous expansion results in larger Da crystals compared to the Dc [29]. However, that relationship was not found here, where a much more isotropic material was formed with a Dc of 112 Å and Da of 97 Å. The more balanced orientation can be attributed to a combination of the higher strain rates and expansion temperature, combined with a larger axial draw ratio than that used in the previous study.

### 3.2. Mechanical Results

#### 3.2.1. Tensile Testing

**Figure 7:**
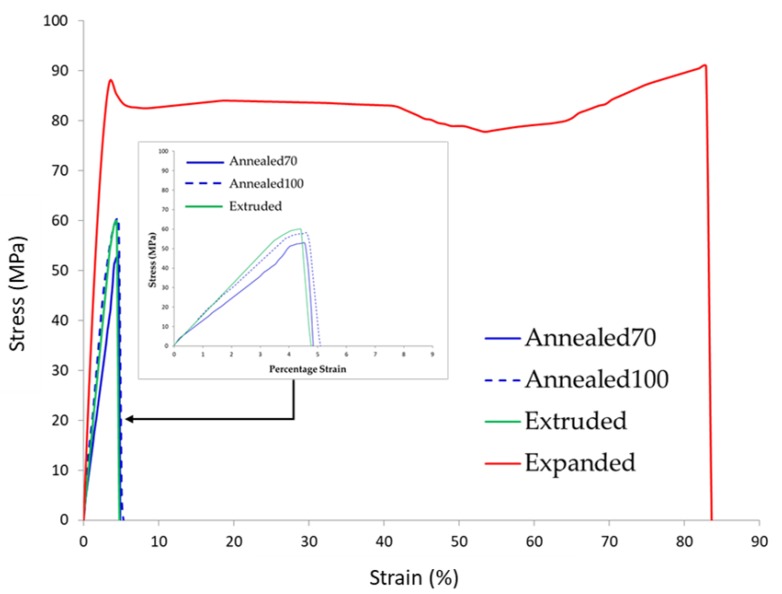
Representative tensile stress/strain curve from Extruded, Annealed70, Annealed100 and Expanded tubing.

Uniaxial tensile testing was performed to assess the impact secondary processing had on the material’s mechanical properties. Figure 7 provides a tensile stress/strain plot with representative samples. The tensile curves for the Annealed70 and Annealed100 samples displayed characteristic brittle behaviour, similar to that of the Extruded tubing. This behaviour was described in a previous study [49], whereby there was no yield point or plastic deformation. Instead, the part failed during elastic deformation, with similar values for maximum load and load at the break.

However, when comparing the stress/strain curve of the biaxially Expanded tubing, it was quite noticeable that the material’s response had changed from brittle to ductile. After initial elastic deformation, the material reached its yield point at a stress of ~90 MPa. This was followed by a sharp decrease in stress, a phenomenon described as strain softening. Initial elastic deformation is caused by straining of the interlamellar amorphous domains, where strain softening is a result of fine chain and course lamella slippages, allowing for a reduction in the force required for plastic deformation [58]. After strain softening, the material underwent necking and plastic deformation until ~65% strain, where it then exhibited a degree of strain hardening before fracture at >80% strain. Strain hardening results from the alignment and orientation of molecular chains and lamellar crystals in the direction of load, causing an increase in the tensile strength and stiffness of the material [59].

Table 2 provides a summary of the recorded tensile properties for Young’s modulus, maximum tensile stress and strain at maximum load. When comparing the tensile properties of the Annealed70, Annealed100 and Expanded tubing to that of the raw Extruded tubing, the following observations were made. There was a 22% and 11% reduction in Young’s modulus for the Annealed70 and Annealed100 samples, respectively, whilst the Expanded tubing saw an increase of 65%. A similar trend was seen with the maximum tensile stress, where there was a reduction in the Annealed70 (19%) and Annealed100 (9.6%) samples, but a 45% increase in the Expanded tubing. In relation to strain at maximum load, there was a negligible increase in the Annealed70 and Annealed100 samples. However, there was a significant change in the Expanded tubing with an 18-fold increase. It should also be noted that there was more variation in the Annealed70, Annealed100 and Expanded tubing results when compared to the Extruded tubing. Extrusion is a continuous process and is therefore more likely to produce material with greater homogeneity. In contrast, temperature gradients inside the annealing oven or small inherent variations in the 1-up biaxial expansion process may have led to larger part to part material variation.

The impact of annealing on the crystallinity and mechanical properties of PLLA has been widely reported [28,31,32,60]. The crystalline regions formed during annealing act as crosslinks giving the polymer higher tensile strength and modulus (stiffness), as compared to its amorphous analogue [61]. However, the results in Table 2 contradict this, with a reduction in both Young’s modulus and maximum tensile stress post-annealing. While the DSC results presented in Table 1 confirmed there was no notable change in crystallinity between the Annealed70 and Extruded tubing, there was a significant increase in the crystallinity of the Annealed100 samples. Therefore, the changes in tensile properties witnessed in the annealed samples are potentially related to stress relaxation and the reduction in orientation associated with annealing, rather than a change in morphology. Heating a polymer above its *T*_g_ provides enough molecular mobility to relieve these residual stresses. The different types of residual stresses found in a semi-crystalline polymer that is cooled from the melt have been described as follows; 1) thermal or cooling stresses owed to differential cooling, 2) orientation resulting in internal stresses, and 3) quenching (physical ageing) stresses caused by cooling the material to quickly below *T*_g_ before thermodynamic equilibrium is achieved. It has also been postulated that annealing above *T*_g_ removes orientation, which again will impact tensile properties [62].

As expected, the biaxial expansion process resulted in a significant increase in all three tensile properties, which can be attributed to a combination of the strain-induced increase in crystallinity and biaxial molecular orientation. Many authors have proposed different mechanisms for the improvement in mechanical properties of PLLA associated with biaxial expansion. One theory associates the improvements with strain-induced amorphous orientation and the packing of crystals, rather than solely related strain-induced crystallinity [29,38]. A separate study, looking at the effect of thermal strain-induced crystallisation of amorphous PLLA, found that the mechanical properties and elongation increased due to the coordination effect of thermally-induced initial crystallinity and moderate chain segment mobility [46]. It has been postulated that the structure consisting of cohesional entanglements formed during melt processing leads to the brittleness of PLLA. Whilst the destruction of this network structure due to disentanglement caused by biaxial stretching leads to the formation of isotropically small crystalline lamellae and an increase in the toughness of PLLA [63]. It’s also believed that molecular orientation during the stretching of PLLA above *T*_g_ results in a change from the brittle fracture mechanism, caused by crazing, to a ductile energy dissipation mechanism. This is attributed to the growing cracks being arrested in the anisotropic structure and postponement of the catastrophic failure [64].

#### 3.2.2. Dynamic Mechanical Analysis (DMA)

DMA testing was performed to assess the impact secondary processing had on the materials’ thermo-mechanical properties. Table 3 provides a summary of storage modulus (*E*’) of the Extruded, Anenaled70, Annealed100 and Expanded tubing at various temperatures below their *T*_g_. Storage modulus relates to the elastic response in a viscoelastic material and is a measure of the materials’ stored energy. The Expanded tubing had an 88.7% higher *E*’ than the Extruded tubing at room temperature (21 °C), indicating a significant increase in the stiffness of the material. This correlated with the tensile results in Table 2, which show the Expanded tubing having a higher Young’s (elastic) modulus and maximum tensile stress than the Extruded tubing. A similar trend was seen in the Annealed100 tubing, with a 75.8% increase in *E*’ at 21 °C when compared to the Extruded tubing. However, the Annealed70 tubing only witnessed a small increase (7.3%) in *E*’. It is noticeable, however, that the 84% difference in Young’s modulus between the Expanded and Annealed100 tubing as reported in Section 3.2.1, was significantly higher than the 7% difference in E’ between both samples. This is most likely due to high strain rates used during tensile testing, not allowing the material enough time to respond to stress with large-scale viscoelastic deformation or yielding [65]. There was also a very subtle drop in modulus between 21 °C (room temperature), 37 °C (body temperature) and 50 °C, which are analogous with the results reported in a previous study [49] and highlight the temperature dependence of mechanical properties below the glass transition temperature in PLLA tubing.

**Figure 8:**
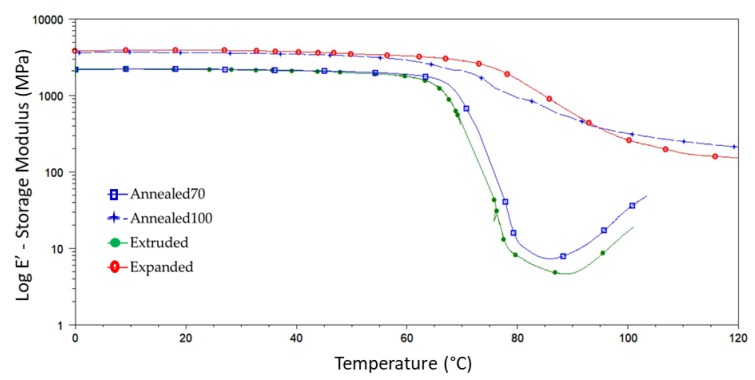
DMA (Dynamic Mechanical Analysis) overlay of the storage modulus plots from the Extruded, Annealed70, Annealed100 and Expanded tubing.

Figure 8 provides an overlay of the storage modulus (*E*’) as a function of temperature for the various samples. The *E*’ for all samples decreased slowly until a sharp drop off could be seen between 55 and 85 °C. As a polymer passes through its *T*_g_, it transitions from a rigid glassy material to a rubbery material. This transition could be seen on a DMA plot by the steep drop in *E*’, whereby the intensity of the drop is indicative of the amorphous fraction of the polymer [34]. It was also clearly seen that the drop in *E*’ was lower in the Annealed100 and Expanded tubes when compared to the Extruded and Annealed70 tubing. This can be attributed to the higher percentage of crystallinity [25,66]. It could also be observed that the E’ started to increase once again between 85–100 °C, on the Extruded and Annealed70 samples. This increase in E’ correlated well with the cold crystallisation reported in the DSC thermograms.

**Figure 9:**
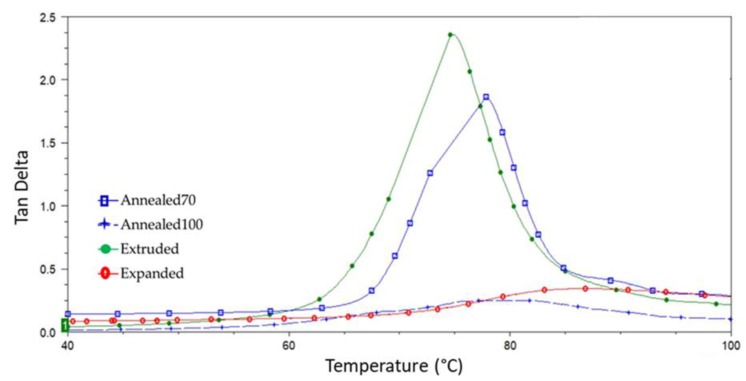
DMA (Dynamic Mechanical Analysis) overlay of the tan delta plots for the Extruded, Annealed70, Annealed100 and Expanded tubing.

Figure 9 represents a plot of the tan delta (tan δ) for each sample. The tan δ is the ratio of storage and loss moduli (tan δ = E’/E’’) and is described as the damping coefficient. The height of the tan δ peaks correlates to differences in the polymer crystallinity, due to the association of the glass transition with the mobility of the polymers amorphous regions [60]. There was a significant reduction in the tan δ peak of the Annealed100 and Expanded tubing, again owed to the increased crystallinity of the material.

## 4. Conclusions

The aim of this study was to directly compare the influence of both annealing and biaxial expansion on the properties of PLLA tubing. As expected, there was very little change in the X_c_ of the tubing annealed at 70 °C for 1 h (Annealed70). However, annealing at a temperature of 100 °C for 1 h (Annealed100) saw the X_c_ increase from ~14% up to 42.2% and the elimination of cold crystallisation. The 2D-WAXS patterns of the Annealed100 tube highlighted that the preferred orientation was parallel to the circumferential direction, which is transverse to the draw direction from the original extrusion process. While DMA testing of the Annealed100 tubing showed an increase in storage modulus, there was no increase in the materials’ ductility.

The biaxial expansion process had the most profound impact on the properties of PLLA tubing. The expansion process resulted in the elimination of cold crystallisation and melt recrystallisation, with crystallinity increasing to 49.9%. The 2D-WAXS patterns showed the emergence of two additional equatorial diffraction peaks in the axial direction. The peaks were owed to the radial expansion of the tube, albeit the peaks were not fully parallel to the axial direction as expected. This tilting phenomenon has been attributed to the high draw temperatures, resulting in a tilting of the crystal chain axis away from the draw direction. These results indicate that the biaxially expanding PLLA at a temperature above *T*_cc_ is possible at the high strain rates associated with stretch blow moulding. The most significant difference between the two secondary processing techniques was their effect on the material’s mechanical properties. The Expanded tubing showed a 65% increase in Young’s modulus, a 45% increase in maximum tensile stress and an 18-fold increase in percentage strain at maximum load compared to the Extruded tubing. In conclusion, it was observed that whilst both secondary processes resulted in increased crystallinity and improved mechanical properties, the biaxial expansion had by far the most significant impact and, therefore, provides a better solution for tailoring the properties of PLLA tubing for medical applications.

## Figures and Tables

**Table 1:** Summary of the thermal properties and degree of crystallinity from each group, included are *T*_g_ (glass transition temperature), Δ*C*_p_ (specific heat change), *T*_cc_ (cold crystallisation), Δ*H*_c_ (enthalpy of cold crystallisation), *T*_mc_ (melting recrystallisation), *T*_m_ (melting temperature), Δ*H*_m_ (enthalpy of melting) and *X*_c_ (degree of crystallinity).

Sample		*T*_g_ (°C)	Δ*C*_p_ J/(g °C)	*T*_cc_ (°C)	Δ*H*_c_ (J/g)	*T*_mc_ (°C)	*T*_m_ (°C)	Δ*H*_m_ (J/g)	*X*_c_ (%)
Extruded	$\bar{X}$ (σ)	61.5 *(0.44)*	0.92 *(0.101)*	100.5 *(0.89)*	28.2 *(1.37)*	159.6 *(0.46)*	180.8 *(0.33)*	40.4 *(2.18)*	13.1 *(0.89)*
Annealed70	$\bar{X}$ (σ)	61.2 *(0.41)*	0.41 *(0.052)*	101.4 *(1.49)*	25.2 *(2.09)*	160.2 *(0.56)*	181.2 *(0.62)*	38.4 *(2.66)*	14.2 *(0.65)*
Annealed100	$\bar{X}$ (σ)	63.2 *(4.65)*	0.37 *(0.225)*	-	-	161.3 *(0.35)*	180.3 *(0.53)*	39.2 *(1.52)*	42.2 *(1.63)*
Expanded	$\bar{X}$ (σ)	74.1 *(1.66)*	0.28 *(0.041)*	-	-	-	180.6 *(0.43)*	46.4 *(2.01)*	49.9 *(2.17)*

**Table 2:** Summary of tensile properties from Extruded, Annealed70, Annealed100 and Expanded tubing.

Sample		Young’s Modulus (MPa)	Maximum Tensile Stress (MPa)	Strain at Maximum Load (%)
Extruded	$\bar{X}$ (σ)	2408 *(66.6)*	66.6 *(1.37)*	4.47 *(0.288)*
Annealed70	$\bar{X}$ (σ)	1881 *(253.2)*	53.9 *(2.02)*	4.88 *(1.018)*
Annealed100	$\bar{X}$ (σ)	2151 *(358.5)*	60.2 *(2.60)*	4.51 *(1.193)*
Expanded	$\bar{X}$ (σ)	3962 *(227.8)*	96.6 *(4.26)*	80.89 *(5.986)*

**Table 3:** DMA (Dynamic Mechanical Analysis) Storage Moduli at different temperatures for the Extruded, Annealed70, Annealed100 and Expanded tubing.

Sample		Storage Modulus (MPa) @ 21 °C	Storage Modulus (MPa) @ 37 °C	Storage Modulus (MPa) @ 50 °C
Extruded	$\bar{X}$ (σ)	2067 *(188.8)*	1984 *(197.3)*	1831 *(234.8)*
Annealed70	$\bar{X}$ (σ)	2218 *(257.5)*	2147 *(212.1)*	2045 *(241.2)*
Annealed100	$\bar{X}$ (σ)	3634 *(302.1)*	3503 *(317.2)*	3281 *(354.6)*
Expanded	$\bar{X}$ (σ)	3900 *(337.6)*	3771 *(357.0)*	3482 *(339.8)*
